# Features of Memory-Like and PD-1^+^ Human NK Cell Subsets

**DOI:** 10.3389/fimmu.2016.00351

**Published:** 2016-09-14

**Authors:** Mariella Della Chiesa, Silvia Pesce, Letizia Muccio, Simona Carlomagno, Simona Sivori, Alessandro Moretta, Emanuela Marcenaro

**Affiliations:** ^1^Dipartimento di Medicina Sperimentale, Università degli Studi di Genova, Genova, Italy; ^2^CEBR, Università degli Studi di Genova, Genova, Italy

**Keywords:** human NK cells, NKG2C, PD-1, memory, HCMV, immune checkpoint, CD57

## Abstract

Human NK cells are distinguished into CD56^bright^CD16^−^ cells and CD56^dim^CD16^+^ cells. These two subsets are conventionally associated with differential functional outcomes and are heterogeneous with respect to the expression of KIR and CD94/NKG2 heterodimers that represent the two major types of HLA-class I-specific receptors. Recent studies indicated that immature CD56^bright^ NK cells, homogeneously expressing the inhibitory CD94/NKG2A receptor, are precursors of CD56^dim^ NK cells that, in turn, during their process of differentiation, lose expression of CD94/NKG2A and subsequentially acquire inhibitory KIRs and LIR-1. The terminally differentiated phenotype of CD56^dim^ cells is marked by the expression of the CD57 molecule that is associated with poor responsiveness to cytokine stimulation, but retained cytolytic capacity. Remarkably, this NKG2A^−^KIR^+^LIR-1^+^CD57^+^CD56^dim^ NK cell subset when derived from individuals previously exposed to pathogens, such as human cytomegalovirus (HCMV), may contain “memory-like” NK cells. These cells are generally characterized by an upregulation of the activating receptor CD94/NKG2C and a downregulation of the inhibitory receptor Siglec-7. The “memory-like” NK cells are persistent over time and display some hallmarks of adaptive immunity, i.e., clonal expansion, more effective antitumor and antiviral immune responses, longevity, as well as given epigenetic modifications. Interestingly, unknown cofactors associated with HCMV infection may induce the onset of a recently identified fully mature NK cell subset, characterized by marked downregulation of the activating receptors NKp30 and NKp46 and by the unexpected expression of the inhibitory PD-1 receptor. This phenotype correlates with an impaired antitumor NK cell activity that can be partially restored by antibody-mediated disruption of PD-1/PD-L interaction.

## Introduction

In physiological conditions, human peripheral blood NK cells include different cell subsets corresponding to different stages of NK cell differentiation. These subsets are characterized by the different expression of some receptors and distinct functional capabilities (1, 2).

The two major peripheral blood NK cell subsets are distinguished on the basis of their relative surface expression of CD56 molecule. In particular, CD56^bright^ NK cells (around 10% of peripheral blood NK cells) are CD16 (Fcgamma RIIII)^dim/negative^, CD117/c-kit^positive^ and express the high affinity IL-2Rα chain (CD25), whereas CD56^dim^ NK cells (around 90%) are CD16^bright^ and express only the intermediate affinity IL-2Rβ and γ chains (CD122/132) (3). In addition, CD56^bright^ NK cells are characterized by higher IL18Rα surface expression than CD56^dim^ subset (4). The same NK cell subsets are also characterized by distinct homing properties due to the different surface expression of chemokine receptors: CD56^dim^ NK cells, expressing CXCR1, CX3CR1, and ChemR23, preferentially migrate to inflamed peripheral tissues (5, 6), whereas CD56^bright^ NK cells, thanks to their CCR7 and CD62L expression, preferentially migrate to secondary lymphoid organs (SLOs) (6). Interestingly, recent data indicate that, in some cases, the CD56^dim^ subset may also *de novo* express CCR7 and migrate toward SLOs (7–9).

Differently from CD56^dim^ NK cells, CD56^bright^ NK cells are characterized by low expression of lytic granules and by production of high amounts of cytokines, such as IFN-γ, TNF-α, and GM-CSF (10, 11). Thus, CD56^bright^ NK cells have been usually considered as “regulatory NK cells” and CD56^dim^ NK cells as “cytotoxic NK cells” (notably CD56^dim^ NK cells can also release large amounts of cytokines but only upon receptor-mediated triggering) (12).

These two NK cell subsets also differ in terms of surface expression of HLA-I-specific receptors. Indeed, CD56^bright^ NK cells express only CD94/NKG2A, whereas CD56^dim^ NK cells may also express KIRs, and/or LIR-1 (13, 14). Since inhibitory and activating receptors can be distinguished within the KIR family (15), two broad groups of KIR haplotypes have been identified on the basis of gene content. A haplotypes express only one activating KIR whereas B haplotypes up to five (16). Also CD94/NKG2A has an activating counterpart represented by CD94/NKG2C (17).

In this context, several studies indicated that CD56^dim^ KIR^+^ NK cells derive from CD56^bright^ KIR^−^ NKG2A^+^ NK cells and that late stages of NK cells maturation are associated with the expression of CD57. This molecule is expressed on a fraction of CD56^dim^ NK cells and is believed to mark a subpopulation of terminally differentiated NK cells that are mainly characterized by the KIR^+^, LIR-1^+^, and CD94/NKG2A^−^ phenotype (18, 19).

In addition to CD56^bright^ and CD56^dim^ NK cell subsets, low frequencies of CD56^neg^ CD16^bright^ NK cells are also detected in healthy donors. In patients with chronic viral infections, this CD56^neg^ NK cell subset expands and a pathological redistribution of the various NK cell subsets occurs. Indeed increments in the percent of CD56^neg^ NK cells have been reported in several pathological conditions, including hepatitis C virus (HCV) (20, 21), human cytomegalovirus (HCMV) (22), hantavirus infections (23), and autoimmune disorders (24–26).

The fact that the CD56^dim^ NK cell subset is often heterogeneous in terms of expression levels of natural cytotoxicity receptors (NCRs: NKp46, NKp30, and NKp44) (27) led to the distinction of two additional NK cell subsets termed NCR^dull^ and NCR^bright^ (28). The demonstration that the NCR surface density correlates with the magnitude of the NK-mediated natural cytotoxicity provided a rational explanation for the clonal heterogeneity of NK cells in killing autologous or allogeneic NK-susceptible targets.

In this context, it is important to consider that, in healthy donors, most CD56^dim^ KIR^+^NKG2A^−^CD57^+^ NK cells are characterized by a lower surface expression of NCRs (18, 19). On the other hand, CD56^bright^ NK cells are characterized by higher NKp46 surface expression as compared to CD56^dim^ NK cells.

Finally, despite the fact that NK cells have always been considered members of the innate immune system, new increasing evidences suggest that NK cells can display some features that are usually attributed to adaptive immune cells, such as expansion and contraction of subsets, increased longevity, and a more potent response upon secondary challenge with the same antigen (memory-like properties) (29).

## Memory-Like NK Cell Subsets Emerging upon HCMV Infection

**Figure 1:**
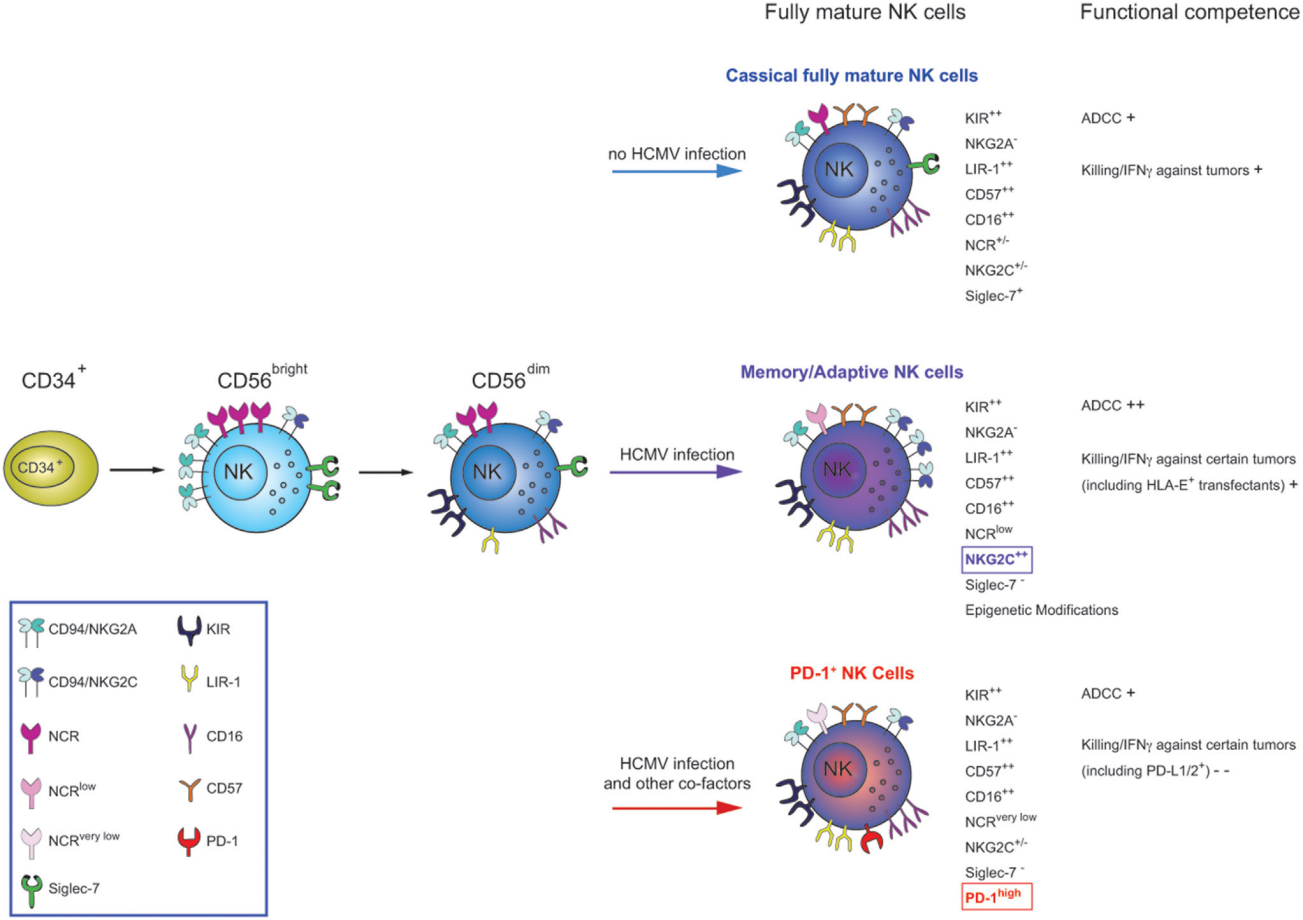
**NK cells differentiate starting from CD34^+^ bone marrow precursors into CD56^bright^ (CD94/NKG2A^+^KIR^−^) and then to CD56^dim^ cells**. CD56^dim^ NK cells, in turn, continue to differentiate throughout their life-span, acquiring novel functional and phenotypic properties. During this process, they lose expression of CD94/NKG2A, sequentially acquire inhibitory KIRs and, at the final step, CD57. This molecule appear to define a subpopulation of highly differentiated NK cells (classical fully mature NK cells), characterized by the KIR^+^LIR-1^+^NKG2A^−^NCR^+/−^ phenotype. Functionally, these cells display natural cytolytic activity and ADCC against tumor targets, but poor responsiveness to cytokine stimulation. After HCMV infection/reactivation, increased proportions of a subset of terminally differentiated CD57^+^ NK cells, characterized by high expression of NKG2C and downregulation of Siglec-7 receptors are induced (the so-called memory-like NK cells). These cells display increased functional capability in terms of ADCC and IFN-γ production/killing in response to HLA-E^+^ and opsonized HCMV-infected targets but decreased function after cytokine stimulation. Following HCMV infections accompanied by other cofactors (infections?), an additional type of CD57^+^ NK cell subset can be generated. This subset is characterized by the expression of the inhibitory PD-1 receptor (not necessary co-expressed with NKG2C) and a very low expression of the NCRs, NKp30 and NKp46. These cells, called PD-1^+^ NK cells, are characterized by compromised effector functions against tumor cells expressing ligands for PD-1 as well as against tumors primarily killed on NCRs/NCR-ligands interactions.

In the last years, it has been observed how HCMV infection can shape the NK cell receptor repertoire inducing the expansion of a specific NK cell population expressing the activating receptor CD94/NKG2C (30, 31) and the marker of terminal differentiation CD57 (32). This HCMV-induced NKG2C^+^CD57^+^ NK cell subset displays a highly differentiated surface phenotype, CD56^dim^CD16^bright^LIR-1^+^KIR^+^NKG2A^−^, and is characterized by the expression of self KIRs (33). More recently, it has been proposed that, upon HCMV infection, NK cells might acquire some hallmarks of adaptive immunity, i.e., clonal expansion, enhanced effector function, longevity, as well as given epigenetic modifications (34–36). Indeed, in HCMV seropositive healthy individuals (HD), the memory-like NKG2C^+^CD57^+^NK cell subset is characterized by an epigenetic remodeling at the IFN-γ locus similar to that found in memory T cells, which is likely responsible for the enhanced IFN-γ production upon target recognition observed in NKG2C^+^ NK cells (37). Interestingly, the HCMV-induced NKG2C^+^ subset is also characterized by a decreased expression of certain signaling molecules, i.e., the adaptor protein FcεRγ and the tyrosine kinase Syk, and by lower expression levels of the transcription factor PLZF, which is involved in regulating epigenetic modifications (e.g., DNA methylation) (34, 35). While the higher accessibility of the IFN-γ locus can enhance IFN-γ production upon target recognition, the lack of FcεRγ could favor a more efficient killing *via* ADCC of opsonized HCMV-infected targets by memory-like NKG2C^+^ NK cells as compared to conventional NK cells (Figure 1). Indeed, in the absence of FcεRγ that only bears one immunoreceptor tyrosine-based activatory motif (ITAM) sequence, CD16 engagement would involve exclusively the adaptor protein CD3ζ, which contains three ITAMs, possibly delivering a stronger signal inside the cell (38, 39). Along this line, a crucial role for CD16 engagement by anti-HCMV antibodies has been proposed not only in promoting ADCC and cytokine release by NKG2C^+^ NK cells but also in favoring their preferential expansion (34, 35, 40). Moreover, the effector function triggered by CD16 engagement in adaptive NKG2C^+^ NK cells (i.e., degranulation and cytokine release) can be enhanced by CD2 costimulation, suggesting a synergy between these receptors in regulating anti-HCMV responses (39).

However, although new insights on memory-like (or adaptive) NK cells are continuously collected, the signals responsible for the described epigenetic modifications and protein expression alterations as well as the exact mechanisms regulating the generation of memory-like NKG2C^+^ NK cells are not completely understood.

The studies carried out in immunocompromised individuals undergoing hematopoietic stem cell transplantation (HSCT), where HCMV infection exerts the strongest effect on NK cells skewing, could help clarifying these aspects. The first observations reported that, in adult UCBT recipients, a rapid accumulation of mature CD56^dim^ KIR^+^NKG2A^−^NKG2C^+^ NK cells occurred following HCMV infection (22, 41). This accelerated maturation was usually accompanied by the downregulation of the inhibitory receptor Siglec-7, which, thus, represents a typical hallmark of the anti-HCMV response in NK cells, along with the expansion of cells expressing NKG2C. Notably, in uninfected UCBT recipients, NK cells were characterized by a more immature phenotype (high frequencies of CD56^bright^ NK cells and NKG2A^+^CD56^dim^ NK cells) even at late time points after UCBT. Remarkably, in some HCMV-infected UCBT recipients, a subset of hyporesponsive CD56^−^CD16^bright^ NK cells displaying a mature phenotype was also observed which likely reflected a condition of severely impaired T cell immunity (22, 42). More recently, a remarkable acceleration of NK cell maturation was described also in pediatric patients receiving a type of allograft different from UCBT, i.e., a HLA-haploidentical HSCT, depleted of both α/β^+^ T cells and B cells and containing variable numbers of donor-derived NK cells and γ/δ^+^ T cells (43). In most recipients, HCMV reactivation favored the preferential expansion of highly differentiated NKG2C^+^CD57^+^selfKIR^+^NKG2A^−^Siglec-7^−^NCR^low^IL18Rα^low^ NK cells and their persistence over time. These cells could kill certain tumor targets, release IFN-γ, display efficient reverse ADCC, and could recognize HLA-E^+^ targets through NKG2C (putative receptor for HCMV). On the other hand, they showed an impaired ability to release IFN-γ upon IL-12+IL-18 exposure. The particular signature shown by these HCMV-induced NK cells may suggest their skewing toward an adaptive condition specialized in controlling HCMV (43). The NKG2C^+^CD57^+^ memory-like NK cell subset could also contribute to protect against leukemia relapse (44). Interestingly, an expansion of memory-like NKG2C^+^CD57^+^ NK cells could be observed also in some recipients who did not reactivate HCMV and who received grafts containing high numbers of mature NK cells derived from a HCMV^+^ donor. Thus, donor-derived transplanted NK cells, primed by a previous encounter with HCMV in the donor, could have persisted and proliferated in the recipient in response to a subclinical reactivation, favoring antiviral responses (43, 45).

Notably HCMV-induced memory NK cell subsets could be represented not only by the described NKG2C^+^CD57^+^ population but also by NKG2C^−^ NK cell subsets, expressing activating receptors different from NKG2C, such as activating KIRs, or other still undefined activating receptors (33, 39, 46, 47).

Thus, in the HSCT setting, HCMV clearly reveals as a key driving force regulating the differentiation of functionally and phenotypically skewed NK cell subsets characterized by memory-like properties.

## Human Resting NK Cells Can Express High Levels of PD-1 Receptor

NK cells are believed to play a critical role in the recognition and eradication of tumors by using different killing strategies; however, tumor cells often develop immunosuppressive mechanisms to avoid NK cell-mediated killing, allowing for tumor escape (48, 49).

An improved understanding of the molecular mechanisms involved in tumor recognition and eradication has led to the identification of checkpoint signaling pathways involved in limiting the anticancer immune response.

One of the most critical checkpoint pathways responsible for mediating tumor-induced immune suppression is the programmed death-1 (PD-1) pathway. This receptor, by modulating the duration and the amplitude of physiological immune responses, is capable of promoting tolerance and preventing tissue damage in settings of chronic inflammation, as well as autoimmune pathologies (48, 49). The induction of the PD-1-dependent inhibitory pathway is mediated by the interaction of this receptor with its ligands, PD-L1 and PD-L2 (50).

The constitutive or inducible expression of the PD-1 receptor has been described on both adaptive and innate immune cells, including T, B, and dendritic cells.

In T cells, binding of PD-1 to its ligands inhibits T cell activation, proliferation, and cytokine production and eventually may result in T lymphocyte exhaustion. Thus, tumors and viruses, by expressing PD-1 ligands, have evolved a remarkable mechanism to hijack the PD-1-dependent regulatory mechanism to avoid T cell-mediated surveillance of cancer or infected cells. Remarkably, however, mAb-mediated blockade of PD-1/PD-L interactions, by disrupting the immune checkpoint-based inhibitory pathway, provides an important opportunity to enhance antitumor immunity particularly in the case of tumor antigen-specific T cells (51, 52).

Similar to T cells, NK cells express surface inhibitory receptors that can be targeted in checkpoint blockade strategies, including the HLA-class I-specific KIR family and CD94/NKG2A heterodimer. Blocking KIR/NKG2A–HLA-class I interactions resulted in potent NK cell-mediated antitumor efficacy (53). Phase I/II trials testing human anti-KIR and anti-NKG2A antibody are ongoing (54–57). Regarding PD-1, the antitumor effect of specific antibodies has been always considered to depend mainly on the rescue effect on activated PD-1^+^ tumor-specific T cells recruited in the tumor environment (58). On the contrary, very little was known on the activated PD-1^+^ NK cells expansions that had been seldom reported in patients with certain tumors or chronic viral infections (59–63). In fact, a precise information on the actual function of these cells was not possible due to the low levels of PD-1 expression and the consequent difficulty in distinguishing positive from negative NK cells.

More insights on the expression and function of PD-1 on NK cells could be recently obtained thanks to the demonstration that the PD-1 receptor is brightly expressed on a discrete cell subset of peripheral blood NK cells from one-fourth of otherwise healthy individuals (64).

PD-1^+^ cells are confined to CD56^dim^ NK cells, and (if present) on CD56^neg^ NK cells, whereas the CD56^bright^ cell subset is consistently PD-1^−^. In addition, a remarkable difference exists among donors regarding the size of the PD-1^+^ NK cell subset. Importantly, the analysis at different time points of the size of PD-1^+^ cell subset in given individuals indicated that this population remains substantially stable over time (64).

The fact that only some of the individuals analyzed are characterized by a PD-1^+^ NK cell subset may be the result of given acute or chronic infection affecting only part of the population (an increase in PD-1^+^ lymphocytes has been associated with HCV, HBV, and HIV) (63, 65–68).

Interestingly, our analysis indicates that a direct correlation between HCMV infection and presence of a PD-1^+^ NK cell subset in the healthy donors analyzed could be established. In particular, we found that PD-1^+^ individuals are in all instances seropositive for HCMV and display higher frequencies of NKG2C^+^ and Siglec-7^−^ NK cells.

By comparing the PD-1^+^ and PD-1^−^ NK cell subsets derived from seropositive PD-1^+^ HD, it was possible to show that the PD-1^+^ subset is confined to cells displaying the phenotypic features of fully mature NK cells, characterized homogeneously by the CD56^dim^ KIR^+^LIR-1^+^NKG2A^−^CD57^+^ phenotype (22, 30). Moreover, only a minor fraction of these cells expressed Siglec-7, whereas, unexpectedly, NKG2C was not necessarily co-expressed with PD-1 receptor. These data could indicate that, in addition to HCMV, additional factors (infections?) may contribute to the induction of PD-1 expression (64).

Further phenotypic characterization indicates that the PD-1^+^ NK cell subset, when compared with PD-1^−^ NK cells, has lower expression of NCRs (NKp46 and NKp30). In addition, the comparison between PD-1^+^ and PD-1^−^ NK cells that are contained within the highly differentiated KIR^+^NKG2A^−^CD57^+^ subset showed that the expression of NCRs is maximally reduced in the PD-1^+^ subset (Figure 1).

Functional analysis of PD-1^+^ NK cells indicated that they display a low cytolytic activity and impaired degranulation against tumor targets, even when these cells lack PD-L1/PD-L2 expression (i.e., K562). The impaired degranulation in response to PD-L^neg^ tumor target cells may be a consequence of the defective expression of NCRs, since these target cells may express a series of ligands for activating NK receptors, such as B7-H6 (69, 70). Remarkably, the reduced degranulation of PD-1^+^ cells following interaction with tumor targets expressing PD-1 ligands is reflecting not only the poor NCR-mediated cell activation but also the inhibitory signal mediated by PD-1 upon interaction with PD-L1/PD-L2 expressed on tumor targets. In this context, it is of note that the inhibition of NK cell degranulation induced by PD-1/PD-L interaction on tumor cells could be partially reverted by mAbs specific for PD-L1/PD-L2 (64).

PD-1^+^ NK cells also display an altered capability of releasing IFN-γ and TNF-α cytokines after stimulation with the same tumor targets used in degranulation assays. Finally, in line with previous data on classical CD57^+^ NK cells, PD-1^+^ NK cells appear to represent a population of poorly proliferating cells, rescued to divide only in the presence of high concentrations of microenvironmental cytokines. This suggests that PD-1^+^ NK cells, like CD57^+^CD8^+^ T cells, have a proliferation defect *in vitro* (e.g., lower expression of IL-2Rβ).

Remarkably, PD-1^+^ NK cells are present in higher proportions in the ascites of ovarian-carcinoma patients (71), suggesting their possible induction/enrichment in tumor microenvironment. Also, in this case, the PD-1^+^ NK cell subset of these patients displayed a functional defect against PD-L1/PD-L2^+^ tumor targets. However, disruption of PD-1 receptor/ligands interaction by specific anti-PD-L mAbs restored degranulation against these tumor target cells.

In conclusion, these findings support the notion that PD-1 signaling may inhibit/block not only T lymphocytes-mediated adaptive responses but also NK cell-mediated innate responses (58, 72).

Therefore, it cannot be excluded that PD-1 may represent an inhibitory checkpoint expressed on NK cells in various cancers of different histotype and that this inhibitory receptor may be involved in the impaired antitumor NK cell responses by these patients.

In this context, it should be stressed/emphasized that, while, in conventional NK cells, the effector function against tumors is primarily regulated by the interactions between HLA-class I-specific inhibitory receptors (KIR and CD94/NKG2A) and HLA-class I molecules, in the case of PD-1^+^ NK cells, the simultaneous expression of PD-1 together with given inhibitory HLA-specific receptors may provide an additional level of suppression of NK cell-mediated antitumor responses. In this case, downregulation of HLA-class I molecules on tumor cells may not be sufficient to induce efficient NK cell responses. These, however, could be restored, at least in part, by mAb-mediated disruption of PD-1/PD-L interaction. On the other hand, in case of PD-L^+^ tumors that do not downregulate HLA-class I molecules, it may be necessary the combined blocking of different inhibitory checkpoints by anti-KIRs and anti-PD-1 mAbs.

## Conclusion

In conclusion, recent studies led to the identifications of novel unexpected properties of NK cells, including the generation of fully mature NK cells displaying some functional characteristics that are reminiscent of cells of adaptive immunity. These cells are generally considered “memory-like” and are characterized by the expression of given set of inhibitory checkpoints mainly represented by different KIRs and LIR-1. Memory-like NK cells that have been originally identified in HCMV^+^ individuals are characterized by functional enhancement in terms of ADCC and IFN-γ production, features linked to changes in the expression of multiple intracellular proteins and transcription factors. On the other hand, these “adaptive” NK cells respond weakly to certain tumors (due to the reduced expression of NKp46 and NKp30) and cytokine receptor-based activation compared to classical fully mature NK cells. Recently, a further novel NK cell subset has been identified in HCMV^+^ individuals. This subpopulation, called “PD-1^+^ NK cells,” is mainly composed by fully mature NK cells and displays strongly reduced capacity to kill PD-L^+^ tumor cells, due to the expression on their surface of high levels of the inhibitory checkpoint PD-1 and of very low levels of NCRs. Due to its ineffective antitumor functions, it would be important to better evaluate the conditions that lead to the generation of this subset and to understand its role in health and disease, in particular in patients with advanced cancers.

In this context, drugs blocking PD-1 and its major ligand PD-L1 have shown great promise in treating many different cancer types. However, the focus is currently only on T cell responses. The fact that a fraction of NK cells express PD-1 can open prospects for extending the potential of cancer immunotherapy to this important innate effector cells.

Innovative treatment could be designed to combine innate immune activation with activation of the adaptive immune system (73).

## Author Contributions

All authors listed have made substantial, direct, and intellectual contribution to the work and approved it for publication.

## Conflict of Interest Statement

AM is founder and shareholder of Innate-Pharma (Marseille, France). The remaining authors have no conflicting financial interests.
